# Special Issue on Designing Hydrogels for Controlled Drug Delivery: Guest Editors’ Introduction

**DOI:** 10.3390/pharmaceutics12010057

**Published:** 2020-01-10

**Authors:** Sonia Trombino, Roberta Cassano

**Affiliations:** Department of Pharmacy and Health and Nutrition Sciences, University of Calabria, Arcavacata di Rende, 87036 Cosenza, Italy

Hydrogels have received growing attention in recent years as materials for drug delivery systems (DDS), because they are biocompatible and nontoxic. They consist of three-dimensional, hydrophilic, polymeric networks capable of imbibing large quantities of water or biological fluids, due to the presence of hydrophilic groups, and releasing the drugs entrapped in them through slow diffusion. According to their features, they can be natural or synthetic and classified as neutral or ionic hydrogels, while the network can be made up of linear homopolymers, linear copolymers, and block or graft copolymers. Hydrogels can provide a spatial and temporal control over the release of various therapeutic agents, including both small molecules and macromolecular drugs. They possess modulable physical properties and the capability to protect labile drugs from degradation controlling their release. This special issue fits into this context and has the aim of describing, through seven research papers and two review articles, recent studies concerning the design, preparation and evaluation of the performance of hydrogels for the controlled delivery of drugs.

In the first article Cesar Torres-Luna and collaboarators evaluated the in vitro release of diclofenac sodium, an anionic drug useful for the treatment of pain and eye inflammation, from contact lenses based on poly-2-hydroxyethyl methacrylate hydrogels containing an embedded microemulsion. In particular, the aim of the work was to extend the release duration of the diclofenac sodium [1]. The oil in water microemulsion systems were prepared with two non-ionic surfactants, Brij 97 or Tween 80 and cetalkonium chloride as cationic surfactant. The results indicated that in such systems, microemulsions serves as a diffusion barrier that retards diclofenac sodium release, while cetalkonium chloride further extends drug release due to the ionic interactions between the positively charged-contact lenses and the negatively charged drug. 

In their research paper Afinjuomo et al. have obtained new injectable inulin hydrogels by crosslinking oxidized inulin with adipic acid dihydrazide without the use of a catalyst or initiator [2]. In vitro releases of 5-fluorouracil (5FU) from the various inulin hydrogels was enhanced in acidic conditions (pH 5) respect to physiological ones (pH 7.4). It was also observed an initial burst release followed by a controlled release pattern. In addition, blank gels did not show any appreciable cytotoxicity, whereas 5-FU-loaded hydrogels demonstrated efficacy against a type of colon cancer cells, which further confirms the potential use of these delivery platforms for direct targeting of 5-FU to the colon. 

Lin and collaborators have fabricated mixed heat-sensitive hydrogel systems to support the release of tacrolimus [3], an immunosuppressant agent useful for acute rejection after allograft. The copolymers used, which consist of poloxamer and poly(l-alanine) with l-lysine segments at both ends (P-Lys-Ala-PLX), were able to transport tacrolimus in a gelled form in situ with acceptable biocompatibility, biodegradability, and low gelation concentrations. F-127 Pluronic has been added to formulate a mixed hydrogel system and to maintain adequate drug levels in animals with transplants. In addition to the in situ gelling properties, the system has shown greater encapsulation efficiency, easy administration and applicability to various types of drugs. Therefore, using these mixed hydrogel systems for sustaining delivery of tacrolimus can give important advancements in immunosuppressive therapy.

Mawazi et al. developed a carbamazepine sustained release new dosage form for pediatric use [4]. In particular, the carbamazepine was first prepared as sustained release microparticles, and then these last were embedded in alginate beads, and finally, these beads were suspended in iota carrageenan gel as vehicle. The developed formulations showed the advantages of a suspension formulation such as a flexible dosing and being easy to swallow. It also could overcome the issue of carbamazepine precipitation that was seen in the suspension formulation, which can lead to overdose. Carbamazepine sustained release gel has the potential to make the advantages of a sustained release dosage form to pediatric patients accessible, especially children below six years old who have no current option for such a formulation.

In their study Mamidi and collaborators made poly 4-mercaptophenyl methacrylate-carbon nano-onions (PMPMA-CNOs = f-CNOs) reinforced natural protein (zein) composites (zein /f-CNOs) using the acoustic cavitation technique [5]. The cytotoxicity of hydrogels was tested with osteoblast cells. The results showed good cell viability and cell growth. To explore the efficacy of hydrogels as drug transporters, 5-fluorouracil release was measured under gastric and intestinal pHs. The results showed pH-responsive sustained drug release over 15 days of study, and pH 7.4 showed a more rapid drug release than pH 2.0 and 4.5. All obtained results suggested that zein/f-CNOs hydrogel could be a potential pH-responsive drug transporter for a colon-selective delivery system.

The aim of the work of Fabiano et al. was to study the impact of the surface characteristics of new nanoparticles contained in a thermosensitive hydrogel formulation based on chitosan or its derivatives, on ocular 5-fluorouracil bioavailability [6]. The chitosan derivatives used to prepare nanoparticles were quaternary ammonium-chitosan conjugate (QA-Ch), S-protected derivative thereof (QA-Ch-S-pro), and a sulphobutyl chitosan derivative (SB-Ch). The drug release from hydrogel formulation containing nanoparticles based on QA-Ch or QA-Ch-S-pro was virtually equal, whereas the hydrogel formulation containing nanoparticle based on SB-Ch was significantly slower. The authors demonstrated that negative charges on the nanoparticles surface slowed down 5-FU release from thermosensitive hydrogel formulation based on chitosan or its derivatives while positive charges increased nanoparticles contact with the negatively charged ocular surface. Either resulted in enhanced ocular bioavailability.

With the aim to deliver progesterone, a sex hormone with neuroprotective effects, to the brain, Cardia and coworkers have fabricated, characterized, and tested *in vivo* progesterone-loaded hydrogel nanoparticle formulations [7]. In particular, nanoparticles, loaded with different progesterone concentrations, have been obtained by polyelectrolyte complex formation between trimethyl chitosan and sodium alginate, followed by ionotropic gelation with sodium tripolyphosphate as a cross-linking agent. All formulations showed a mean diameter compatible with inhalable administration and a high progesterone encapsulation efficiency. The zeta potential values were set to ensure nanoparticles stability against aggregation phenomena as well as interaction with negative sialic residues of the nasal mucosa. Finally, in vivo studies on Sprague-Dawley male rats demonstrated a 5-fold increase in brain progesterone concentrations compared to basal progesterone level after 30 min of hydrogel nanoparticle inhalation.

The two review articles treated in this special issue concern the characteristics and performances of two types of hydrogels: the first of natural origin, based on hyaluronic acid and the second type a synthetic one based on poloxamers. In particular, Trombino et al. described the properties of hydrogels made with hyaluronic acid (HA) in the release of drugs [8]. HA is an interesting material for hydrogels design due to its biocompatibility, native biofunctionality, biodegradability, non-immunogenicity, and versatility. In the last years, different strategies for the design of physical and chemical HA hydrogels have been developed, such as click chemistry reactions, enzymatic and disulfide crosslinking, supramolecular assembly via inclusion complexation, and so on. HA-based hydrogels are widely investigated for biomedical applications like drug delivery, tissue engineering, regenerative medicine, cell therapy, and diagnostics. Furthermore, the overexpression of HA receptors on various tumor cells makes these materials promising drug delivery systems for targeted cancer therapy.

Finally, Russo and Villa focused on heat-resistant hydrogels consisting of poloxamers which are of great interest for the administration of ophthalmic, injectable, transdermal and vaginal drugs [9]. The particular characteristic of these hydrogels is that they remain fluid at room temperature and become more viscous gels when exposed to body temperature. In this way, the gelation system remains topical for a long time and the release of the drug is controlled and prolonged. Poloxamers can have different consistencies and be liquids, pastes and solids, with respect to the molecular weights and weight ratios of ethylene oxide-propylene oxide present in their structure. Concentrated aqueous solutions of poloxamers form heat-reversible gels that arouse interest in tissue engineering. Finally, the use of poloxamers as biosurfactants has been described since they are capable of forming micelles in an aqueous environment above a concentration threshold known as critical concentration of micelles. This property is interesting for drug delivery and various therapeutic applications.

The purpose of this special issue was to provide an overview of recent advances in delivery of drugs by synthetic or natural hydrogels. Based on the interesting results described in the research articles and reviews presented here, we can conclude that these types of DDS make a significant contribution to the pharmaceutical field.
